# Identification and Validation of Prognosis-Related Necroptosis Genes for Prognostic Prediction in Hepatocellular Carcinoma

**DOI:** 10.1155/2022/3172099

**Published:** 2022-06-29

**Authors:** Xin Gao, Di Huang, Shu-Guang Li, Wen-Xin Wang, Dong-Lei Sun, Jia-Ming Qian, Xiao-Lan Zhang

**Affiliations:** ^1^Department of Gastroenterology, The Second Hospital of Hebei Medical University, Hebei 050035, China; ^2^Department of Gastrointestinal Oncological Surgery, The First Affiliated Hospital of Hebei North University, Hebei 075000, China; ^3^Department of Gastroenterology, Peking Union Medical College Hospital, Chinese Academy of Medical Sciences, Beijing 100730, China

## Abstract

**Background:**

The prediction of hepatocellular carcinoma (HCC) survival is challenging because of its rapid progression. In recent years, necroptosis was found to be involved in the progression of multiple cancer types. However, the role of necroptosis in HCC remains unclear.

**Methods:**

Clinicopathological parameters and transcriptomic data of 370 HCC patients were obtained from TCGA-LIHC dataset. Prognosis-related necroptosis genes (PRNGs) were identified and utilized to construct a LASSO risk model. The GEO cohorts (GSE54236 and GSE14520) were used for external validation. We evaluated the distribution of HCC patients, the difference in prognosis, and the accuracy of the prognostic prediction of the LASSO risk model. The immune microenvironment and functional enrichment of different risk groups were further clarified. Finally, we performed a drug sensitivity analysis on the PRNGs that constructed the LASSO model and verified their mRNA expression levels in vitro. *Results:* A total of 48 differentially expressed genes were identified, 23 of which were PRNGs. We constructed the LASSO risk model using nine genes: *SQSTM1*, *FLT3*, *HAT1*, *PLK1*, *MYCN*, *KLF9*, *HSP90AA1*, *TARDBP*, and *TNFRSF21*. The outcomes of low-risk patients were considerably better than those of high-risk patients in both the training and validation cohorts. In addition, stronger bile acid metabolism, xenobiotic metabolism, and more active immune cells and immune functions were observed in low-risk patients, and high expressions of *TARDBP*, *PLK1*, and *FLT3* were associated with greater drug sensitivity. With the exception of *FLT3*, the mRNA expression of the other eight genes was verified in Huh7 and 97H cells*. Conclusions*. The PRNG signature provides a novel and effective method for predicting the outcome of HCC as well as potential targets for further research.

## 1. Introduction

Necroptosis is a unique type of inflammatory programmed cell death that occurs when apoptotic pathways are halted or inhibited, and TNF-*α*, Fas ligand, and tumor necrosis factor-related apoptosis-inducing ligand stimulated death receptors—TNFR1, TNFR2, and FAS—are the most prevalent triggers [1]. The role of necroptosis in the progression of cancer is complicated. On the one hand, tumor cells can be eliminated directly by the process of necroptosis [2]. In addition, necroptosis supplies antigens and inflammatory stimuli to dendritic cells to kick-start acquired immunity, which then activates CD8^+^T cells and antitumor immune responses [3]. On the other hand, inflammatory responses caused by cytokines released by necrotic cells can promote the development of tumors [4, 5]. Recently, a prognosis-related necroptosis gene signature was used to predict the prognosis and describe the immune microenvironment in human tumors [6, 7].

Hepatocellular carcinoma (HCC) is one of the most aggressive tumors, and the risk factors predominantly include HBV and HCV infection, alcoholic liver disease, and nonalcoholic fatty liver disease [8]. It has been found that hepatocytes can form different types of tumors (intrahepatic cholangiocarcinoma and HCC), which are mainly determined by the mode of cell death (apoptosis and necroptosis) in the tumor microenvironment [9]. In addition, chronic inflammation is an important driving factor for the development of hepatocellular carcinoma, since the release of damage-associated molecular patterns associated with necroptosis can promote angiogenesis and cell proliferation, thus promoting tumor growth and metastasis [10]. Furthermore, the selection of targeted medicines as well as survival prediction remains major challenges because of the lack of effective molecular prognostic indicators. Therefore, we identified differentially expressed genes (DEGs) associated with necroptosis in this study, which was applied to develop a predictive model and describe immunological features in various modes. Our findings will provide a thorough overview of necroptosis-related genes that may have a role in the development of HCC as well as a novel perspective on the clinical application of immunotherapy drugs.

## 2. Materials and Methods

### 2.1. Datasets

We obtained the transcriptomic expression data and clinicopathological information of 370 HCC patients from The Cancer Genome Atlas-Liver Hepatocellular Carcinoma (TCGA-LIHC) database (https://portal.gdc.cancer.gov/projects/TCGA-LIHC) in October 2021. The same data of 78 and 221 HCC patients were obtained from the two Gene Expression Omnibus cohorts (GEO, ID : GSE54236 and GSE14520, https://www.ncbi.nlm.nih.gov/geo/) respectively.

### 2.2. Analysis of DEGs Associated with Necroptosis

We identified 67 genes associated with necroptosis from a previous study [11], which are presented in Supplementary Table 1. We utilized the “Limma” *R* package to normalize the mRNA expression levels and screened DEGs associated with necroptosis in TCGA-LIHC. The associations of DEGs were obtained by the protein-protein interaction network (PPI network; https://string-db.org/cgi/input.pl), and Cytoscape was used to visualize the results and obtain hub genes. We analyzed the relationship between all DEGs and prognosis using Gene Expression Profiling Interactive Analysis (GEPIA) platform (http://gepia.cancer-pku.cn/) and explored their mutation characteristics using GSCALite platform (http://bioinfo.life.hust.edu.cn/GSCA/).

### 2.3. Clustering Analysis of TCGA-LIHC According to DEGs

We further screened out prognostic DEGs by univariate Cox regression (*p* < 0.05). Based on these DEGs, we performed a cluster analysis (“ConsensusClusterPlus” *R* package) and explored clinicopathological differences between different clusters.

### 2.4. Construction and Validation of the Least Absolute Shrinkage and Selection Operator Regression Model

We used the “GLMnet” *R* package to build the least absolute shrinkage and selection operator (LASSO) regression model. The specific method was described previously [12]. In short, nine genes were used to establish a risk model and divided TCGA-LIHC into high-risk and low-risk groups according to the median risk score. Then, the mRNA levels of the GSE54236 and GSE14520 cohorts were standardized as a validation cohort via the “sva” function in R. The risk score of each HCC patient was calculated according to the same formula. Overall survival (OS) of the two subgroups was compared based on the Kaplan–Meier analysis. Principal component analysis (PCA) and t-distributed stochastic neighbor embedding (t-SNE) analysis were performed by the “t-SNE” package in R. The receiver operating curve (ROC) and area under the curve (AUC) of 1-, 3-, and 5-year OS were analyzed using the “survivalROC” package in *R*.

### 2.5. Functional Enrichment Analysis

Gene set enrichment analysis (GSEA) with h.all.v7.2.symbols gene sets was used to investigate potential biological functions according to risk scores. In addition, we calculated the difference in the immune cell enrichment fraction and function between high- and low-risk groups by single-sample gene set enrichment analysis (ssGSEA) [13].

### 2.6. Cell Culture

The cells and culture conditions were as follows: human normal liver cell line LO-2 (RPMI-1640 containing 10% FBS), and HCC cell lines Huh7 (MEM containing 10% FBS) and 97H (DMEM containing 10% FBS) in a 5% CO_2_ incubator at 37°C. The total RNA was extracted with TRIZOL (Invitrogen) reagent. Then, cDNA was obtained by reverse transcription with cDNA Synthesis Mix (E047-01B; Novoprotein) and analyzed by quantitative PCR (E096-01B; Novoprotein). The mRNA expression levels were normalized against *GAPDH* expression. All primers used for the nine genes in this study are listed in Supplementary Table S2.

### 2.7. Statistical Analysis

All statistical analyses were completed by *R* software 4.0.1, and *p* < 0.05 were considered to be statistically significant.

## 3. Results

### 3.1. Identification of DEGs, Hub Genes, and Mutation Patterns Associated with Necroptosis

**Figure 1:**
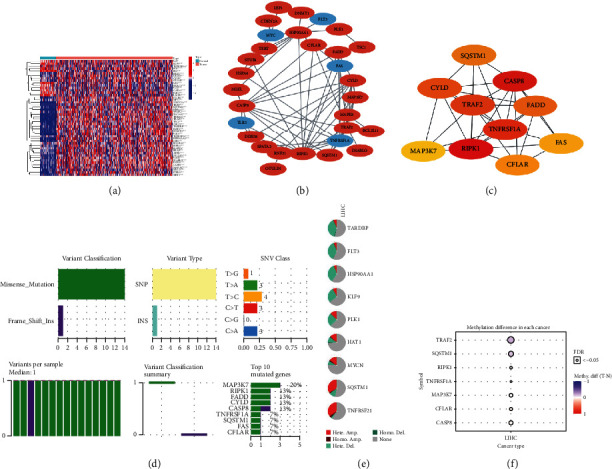
Screening of DEGs in HCC patients from TCGA database. (a) Expression heatmap of DEGs between normal and tumor tissues in TCGA-LIHC cohort. (b) Interaction between DEGs visualized by Cytoscape (red: positive correlation; blue: negative correlation. The depth of the color represents the strength). (c) Identification of hub genes according to the MCC algorithm. Single nucleotide variation (d), copy number alterations (e), and methylation state (f) of hub genes.

The expression levels of a total of 67 genes with necroptosis were calculated, and we identified 10 downregulated genes (*ID1*, *BACH2*, *AXL*, *MYC*, *GATA3*, *TLR3*, *FLT3*, *KLF9*, *TNFRSF1A*, and *FAS*) and 38 upregulated genes (*HDAC9*, *DDX58*, *CYLD*, *MAPK8*, *CFLAR*, *MPG*, *RIPK1*, *TARDBP*, *SIRT2*, *BRAF*, *BCL2L11*, *OTULIN*, *ATRX*, *DIABLO*, *STUB*, *SPATA2*, *MLKL*, *MAP3K7*, *HAT1*, *FADD*, *ITPK1*, *CASP8*, *HSP90AA1*, *RNF31*, *HSPA4*, *USP22*, *TNFRSF21*, *SLC39A7*, *TSC1*, *SQSTM1*, *TRIM11*, *TRAF2*, *DNMT1*, *LEF1*, *PLK1*, *MYCN*, *CDKN2A*, and *TERT*) in tumor tissue compared with normal tissue. The visual results of the DEGs are shown in a heatmap in Figure 1(a). The correlation network is shown in Figure 1(b). A PPI network was used to identify the interactions between DEGs, which was displayed in Cytoscape, and the MCC algorithm in Cytohubba plug-in was used to calculate the top 10 hub genes (Figure 1(c)). A missense mutation was the main single nucleotide mutation type (Figure 1(d)). These genes also had different levels and types of copy number variation (Figure 1(e)) and showed different patterns of methylation (Figure 1(f)).

### 3.2. Relationship Between Hub Genes and Prognosis

We used the GEPIA platform to calculate the relationship between hub genes and prognosis. For OS, *FADD*, *MAP3K7*, and *TNFRSF1A* were associated with worse outcomes (*p* < 0.05) and *CFLAR*, *SQSTM1*, and *RIPK1* showed the same trend. In addition, the high expression of *TRAF2* was associated with shortened disease-free survival (*p* < 0.05), and *CASP8*, *FADD*, and *SQSTM1* showed the same trend (Supplementary Figure 1). Other genes had no significant correlation with prognosis.

### 3.3. HCC classification according to prognostic-related necroptosis genes (PRNGs)

**Figure 2:**
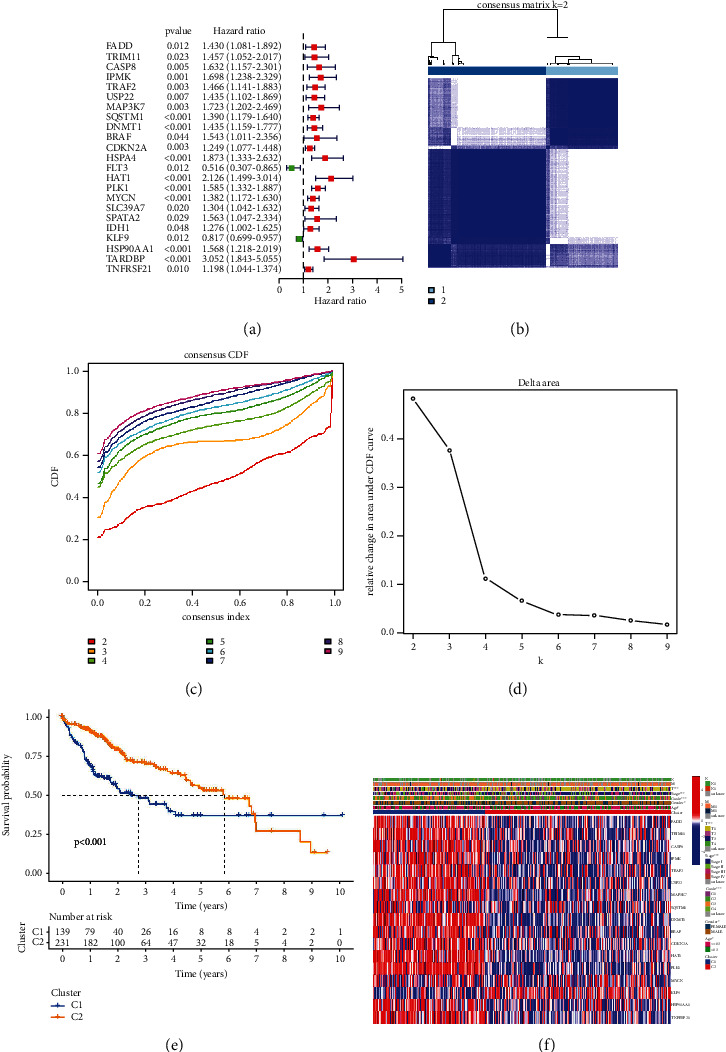
Identification of the molecular subtypes of TCGA-LIHC according to PRNGs. (a) Forest plot of univariate Cox regression analysis according to PRNGs. (b, c, d) TCGA-LIHC patients were divided into two groups based on the consensus clustering matrix (*k* = 2). (e) Kaplan–Meier OS curves in the two clusters. (f) Correlations between the two groups and their clinicopathological characteristics are depicted in a heatmap.

We identified 23 PRNGs using univariate Cox analysis. *KLF9* and *FLT3* were associated with better prognosis (hazard ratio <1), whereas *FADD*, *TRIM11*, *CASP8*, *IPMK*, *TRAF2*, *USP22*, *MAP3K7*, *SQSTM1*, *DNMT1*, *BRAF*, *CDKN2A*, *HSPA4*, *HAT1*, *PLK1*, *MYCN*, *SLC39A7*, *SPATA2*, *IDH1*, *HSP90AA1*, *TARDBP*, and *TNFRSF21* were associated with worse prognosis (hazard ratio >1, Figure 2(a)). We performed a cluster analysis and divided TCGA-LIHC into two categories according to CDF values (Figures 2(b)–2(d)). The survival analysis results showed that cluster 1 had a better prognosis than cluster 2 (Figure 2(e)), and the clinicopathological parameters of the two clusters were significantly different, which suggested that different clinical features represent different necroptosis patterns (Figure 2(f)).

### 3.4. Establishment and Validation of a PRNG LASSO Risk Model in TCGA Training Cohort and GEO Test Cohorts

**Figure 3:**
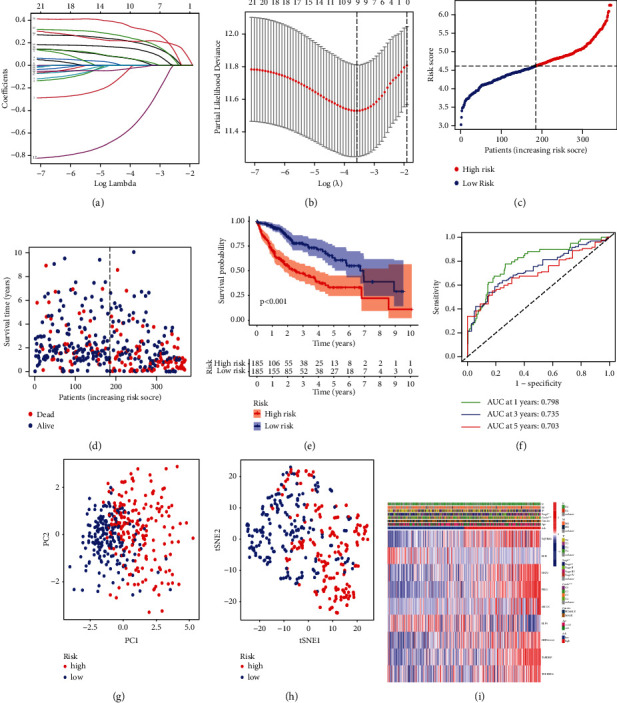
Construction of a LASSO risk model based on TCGA-LICH training cohort. (a, b) Construction of the LASSO regression model according to the 23 PRNGs using TCGA-LIHC training cohort. (c) Distribution of the risk scores. (d, e) High-risk patients had higher mortality rates. (f) ROC analysis. (g, h) PCA and t-SNE analysis. (i) Correlations between the two subgroups and their clinicopathological characteristics are depicted in a heatmap.

We performed LASSO regression analysis using 23 PRNGs in TCGA-LIHC training cohort to establish the prognostic model. To minimize overfitting, nine genes were used to generate the final TCGA LASSO risk model (Figures 3(a) and 3(b)), and the risk genes and their coefficients are shown in Table 1. The formula used to calculate risk scores was as follows: Sum of (gene expression^*∗*^coefficient). Based on the median risk score, TCGA-LIHC patients were divided into high- and low-risk groups (Figure 3(c)). There were more deaths and poorer OS in the high-risk group (Figures 3(d) and 3(e)). The subsequent ROC analysis revealed that the risk model could accurately assess and predict the survival of HCC patients (AUC at 1, 3, and 5 years was 0.789, 0.735, and 0.703, respectively; Figure 3(f)). Finally, the PCA plot and t-SNE plot revealed that the risk models could distinguish the high- and low-risk groups to some extent (Figures 3(g) and 3(h)). Heatmaps showed worse staging and pathological grade in the high-risk group (Figure 3(i)).

**Figure 4:**
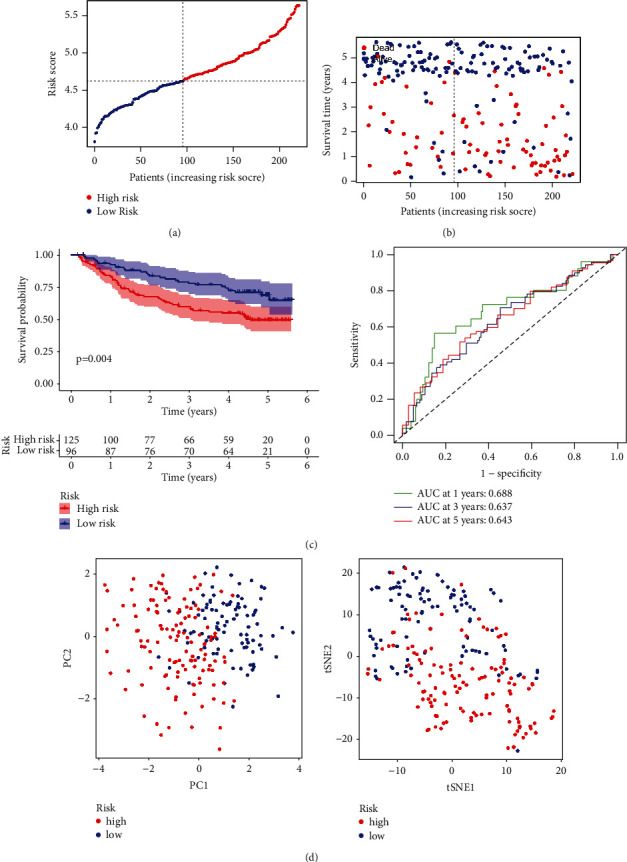
Validation of the LASSO risk model based on the GSE14520 test cohort. (a) Distribution according to the risk scores. (b) Low-risk patients have favorable overall survival. (c) The prognosis of low-risk patients is better than that of high-risk patients, indicating that the risk model has a certain predictive value. (d) Two groups of HCC patients can be better distinguished according to PCA and t-SNE analysis.

The prognostic assessment of the risk model was well reproduced in the GEO validation cohort. We first used SE14520 to validate the risk model. The risk scores of 221 HCC patients were calculated according to the previous formula, and 96 patients were assigned to the low-risk group and 125 to the high-risk group (Figure 4(a)). Patients in the low-risk group had longer OS (Figures 4(b) and 4(c)). The AUC of 1-year, 3-year, and 5-year OS was 0.688, 0.637, and 0.643, respectively (Figure 4(c)), and the PCA plot and t-SNE plot revealed that the risk genes were effective in distinguishing between the two risk groups (Figure 4(d)). The GSE54236 cohort containing 78 HCC patients showed similar results, and the AUC of 1-year and 3-year OS was 0.665 and 0.631, respectively (Supplementary Figure 2).

### 3.5. Independent Prognostic Analysis and Establishment of a Prognostic Nomogram Based on TCGA-LIHC

**Figure 5:**
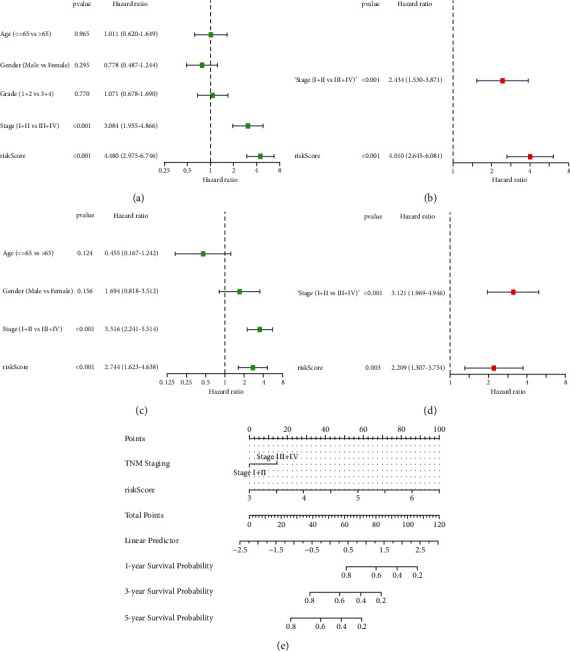
Independent prognostic analysis and nomogram. (a) Univariate and (b) multivariate Cox analyses of TCGA-LIHC training cohort. (c, d) Risk score was also an independent prognostic risk factor for the validation cohort. (e) Nomogram incorporating a risk score that can be used to forecast OS in HCC patients.

We further evaluated whether the risk model could be used as an independent predictor of prognosis. For the training cohort, TNM staging (*p* < 0.001, HR = 3.084, 95% CI: 1.955–4.866) and risk score (*p* < 0.001, HR = 4.480, 95% CI: 2.975–6.746) were possible risk factors in the univariate Cox regression analysis (Figure 5(a)). In the multivariate analysis, the risk score was an independent prognostic factor (*p* < 0.001, HR = 4.010, 95% CI: 2.645–6.081; Figure 5(b)). For the GSE14520 cohort, risk score is also an independent prognostic factor (*p*=0.003, HR = 2.209, 95% CI: 1.307–3.734; Figures 5(c) and 5(d)). Finally, we created a novel prognostic nomogram based on the training cohort that incorporates risk scores and clinical data to provide a credible method for predicting HCC patient survival (Figure 5(e)).

### 3.6. Functional Enrichment Analysis

**Figure 6:**
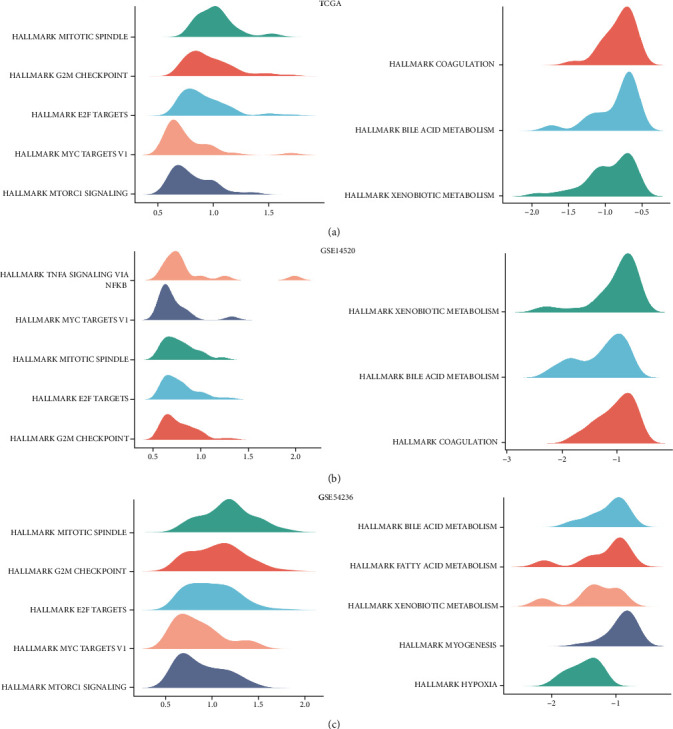
The results of GSEA were similar to the three cohorts. (a) TCGA-LIHC training cohort. (b) GSE14520 validation cohort. (c) GSE54236 validation cohort.

We further attained DEGs (logFC >0.585,*p* < 0.05) to distinguish the biological functions and networks associated with the risk group in the training and validation cohorts. Total GSEA analysis results are presented in Supplementary Table 3.  Figure 6(a) shows that the top five hallmarks, SPERMATOGENESIS, MITOTIC_SPINDLE, G2M_CHECKPOINT, E2F_TARGETS, and MYC_TARGETS_V1, were associated with the high-risk subgroup, while three hallmarks, COAGULATION, BILE_ACID_METABOLISM, and XENOBIOTIC_METABOLISM, were more enriched in the low-risk subgroup in the training cohort. The enrichment analysis results of the two validation cohorts were similar to the training cohort (Figures 6(b) and 6(c)).

### 3.7. Differences in the Tumor Immune Microenvironment Associated with Risk Subgroups

**Figure 7:**
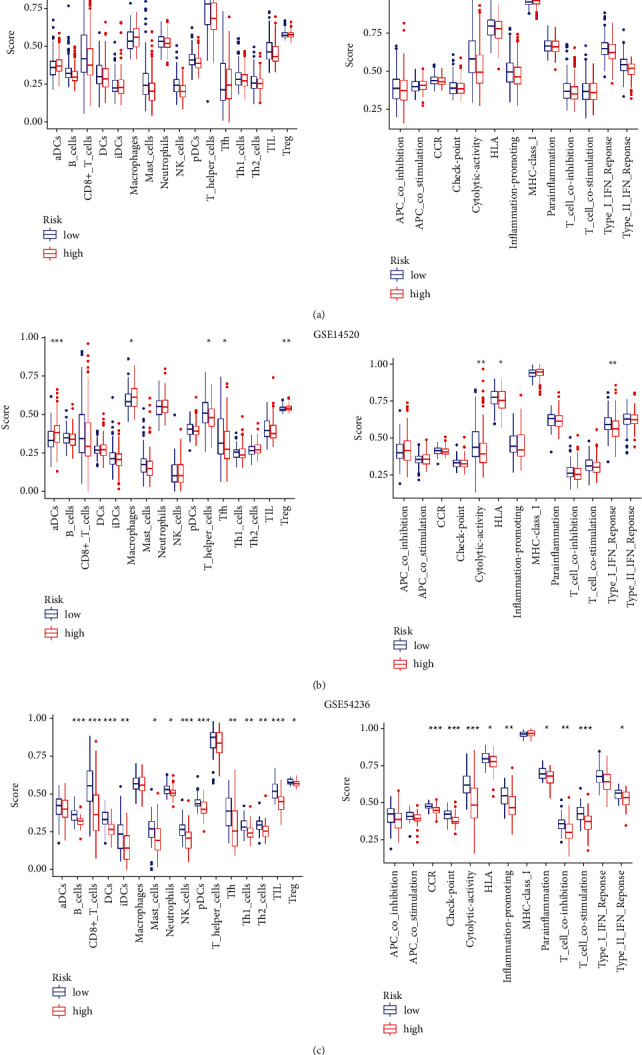
Both the training cohort and the validation cohorts revealed richer immune cell infiltration and immune function in low-risk patients. (a) TCGA-LIHC training cohort. (b) GSE14520 validation cohort. (c) GSE54236 validation cohort. (^*∗*^*p* < 0.05; ^*∗∗*^*p* < 0.01; ^*∗∗∗*^*p* < 0.001).

Despite the lack of effective immunotherapeutic biomarkers for HCC, the immune score of the tumor immune microenvironment is a promising indicator. Therefore, we further investigated 16 types of immune cells and 13 types of immune functions in different risk subgroups according to ssGSEA. We found that low-risk patients had more activated immune cells and functions in both the training cohort and the validation cohorts (Figure 7), which may be the reason for the better prognosis of low-risk patients.

### 3.8. Relationship Between Risk Model Genes and Drug Sensitivity

**Figure 8:**
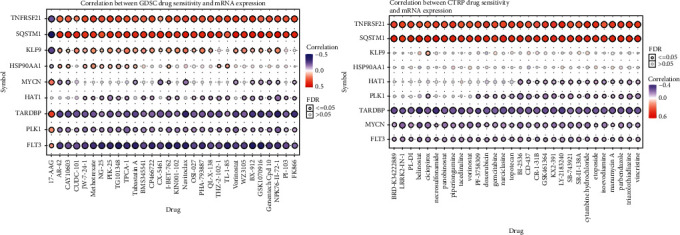
Correlation between drug sensitivity and risk gene expression according to the GDSC and CTRP databases.

By analyzing the GDSC and CTRP databases, potential drugs were found to be associated with genes involved in the risk model. In general, we found that high expression of *TNFRSF21* and *SQSTM1* mostly reduced drug sensitivity, while the high expression of *TARDBP*, *PLK1*, and *FLT3* enhanced drug sensitivity (Figure 8).

### 3.9. Validation of the Expression of Risk Model Genes

**Figure 9:**
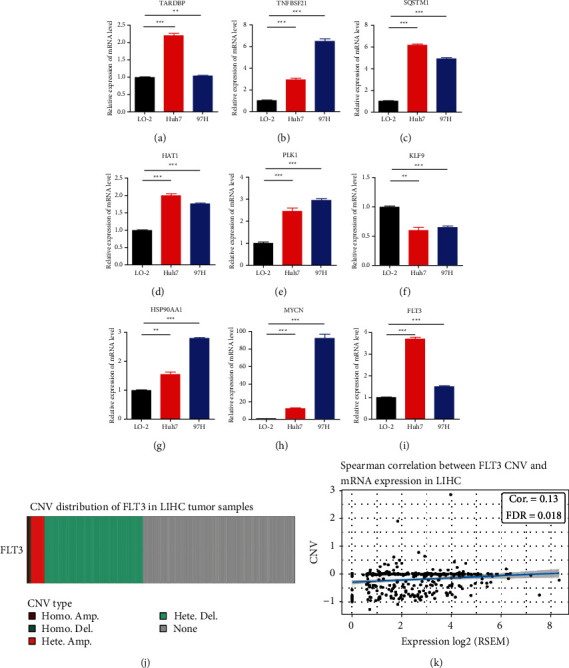
mRNA expression in vitro validation experiment CNV characteristics of *FLT3*. (a-i) mRNA expression of nine risk model genes in normal liver and HCC cell lines. (j) Copy number deletions in *FLT3*. (k) Copy number was positively correlated with mRNA expression. (^*∗*^*p* < 0.05; ^*∗∗*^*p* < 0.01; ^*∗∗∗*^*p* < 0.001).

We compared the mRNA levels of the risk model genes in two HCC cell lines (Huh7 and 97H) and a normal liver cell line (LO-2). The expression of all the other genes was consistent with the previous results except for the increased expression of *FLT3* in tumor cell lines (Figures 9(a)–9(i)). It is reported that FLT3 promotes the proliferation and migration of HCC, so we further investigated the reasons for the decrease in FLT3 expression. The results showed that *FLT3* copy number deletion mutation exists in 37.5% of patients in TCGA-LIHC which is related to the level of mRNA expression (Figures 9(j)–9(k)).

**Figure 10:**
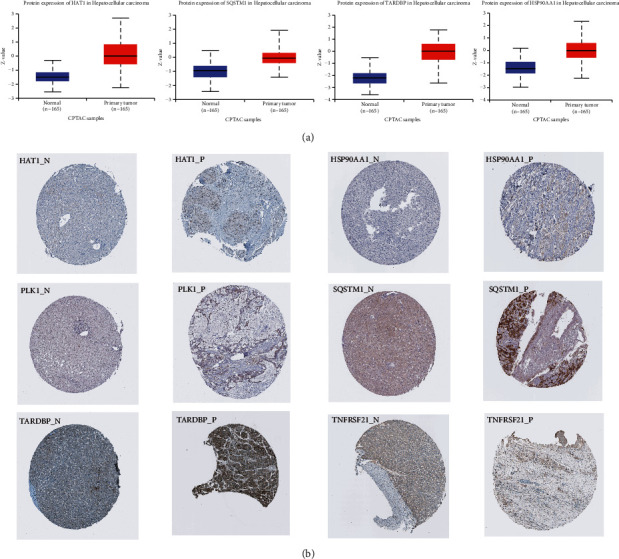
Protein expression of nine risk model genes between HCC and normal samples. (a) Protein expression levels of HAT1, SQSTM1, TARDBP, PLK1, and HSP90AA1 in the CPTAC database. (b) Immunohistochemical results of HAT1, SQSTM1, PLK1, and HSP90AA1 in the normal and tumor groups in the HPA database.

In addition, protein expression levels of risk model genes (HAT1, SQSTM1, TARDBP, and HSP90AA1) were obtained from the CPTAC database (Figure 10(a)). Meanwhile, we verified the protein expression levels of HAT1, SQSTM1, PLK1, HSP90AA1, TARDBP, and TNFRSF21 using the HPA database (Figure 10(b)).

## 4. Discussion

HCC is the second most lethal tumor after lung cancer, and there were approximately 830,180 new deaths worldwide in 2020 [14]. In recent years, the traditional prognostic evaluation system based on clinicopathological parameters and staging has not been able to meet the requirements of precision medicine [15]. With the development of sequencing technology, researchers have paid more attention to the molecular typing of diseases and the seeking of new biomarkers to guide clinical diagnosis and treatment [16]. This strategy not only complements the traditional prognosis evaluation, but also reveals a new pathogenesis. Necroptosis is a unique way of cell death. Recently, its characteristics have been described in a variety of human tumors. Generally, according to different subtypes of necroptosis, the prognosis of patients can be accurately predicted [6, 7]. In hepatocellular carcinoma, it has been recognized that necroptosis is a double-edged sword that provides the inflammatory environment required for carcinogenesis, while the immune response is launched to fight against tumors [17]. However, the signature of necroptosis genes has not been fully described in HCC.

This study systematically identified DEGs related to necroptosis in patients with HCC. First, we found that 10 genes were downregulated and 38 genes were upregulated in tumor samples. Then, we performed consistent clustering according to 23 PRNGs and found that patients with HCC could be divided into two subtypes between which the survival time differed substantially. Interestingly, the clusters were associated with clinicopathological parameters, which means that the strong inflammation caused by necroptosis compels tumor cells face severe natural selection, which leads to stronger invasiveness of some subclones and poor prognosis. Subsequently, nine genes were used to build a LASSO risk model according to the training cohort, which was well verified in the test cohorts. We found that risk score was an independent prognostic factor and mapped a nomogram to predict the OS of HCC patients. We further illuminated the functional enrichment characteristics of different risk subgroups. Mitotic spindle disruption [18], E2F [19], MYC pathways [20], mTORC1 signaling [21], and G2/M cell cycle [22] enriched in the high-risk group are all associated with the stronger invasiveness of HCC.

HCC is characterized by low tumor mutational burden and microsatellite stability, and the expression of immune checkpoints fails to predict the response of patients to immunotherapy [23]. Therefore, evaluation of the tumor immune microenvironment may be the critical index of immunotherapy in the future [24]. Recent studies revealed that immune checkpoint inhibitors cannot activate exhausted T cells, and the characteristic of “hot” tumor (innate attraction of T cells) is the key to the effectiveness of immunotherapy [25, 26]. Conceivably, the low-risk patients may benefit from immunotherapy due to more active immune cells and a stronger immune function. In addition, we also conducted drug sensitivity analysis and found that high expression of *TNFRSF21* and *SQSTM1* mostly reduced drug sensitivity, while high expression of *TARDBP*, *PLK1*, and *FLT3* enhanced drug sensitivity.

*SQSTM1*, *TNFRSF21*, *TARDBP*, *HAT1*, *PLK1*, *MYCN*, *KLF9*, *HSP90AA1*, and *FLT3* were applied in the construction of the LASSO risk model. SQSTM1 is reported as an autophagy receptor that can serve as a bridge between polyubiquitinated cargo and autophagosomes as well as mediate necroptosis by recruiting RIPK1 [27]. SQSTM1, TNFRSF21, and TARDBP have rarely been reported in HCC, but they are associated with poor outcomes (Figure 2). HAT1 is an acetyltransferase that can form a complex with RIP1/3 to reduce programmed cell death [28]. PLK1 can activate the NF-*κ*B signaling pathway to promote HCC development; thereby, harnessing necroptosis through inhibiting PLK1 may be a promising treatment strategy [29, 30]. MYCN [31] and HSP90AA1 [32] can promote HCC by activating the MYC pathway.

*FLT3* was the only gene in our validation experiment that was inconsistent with the results of the bioinformatic analysis. *FLT3* belongs to the receptor tyrosine kinase family, which is more widely reported in hematological diseases [33]. Sorafenib is the first-line drug for HCC and is a multi-kinase inhibitor, targeting FLT3 among others. The previous results showed that the mRNA expression of *FLT3* was decreased in 64% of patients with HCC because of the loss of gene copy number; however, patients with high *FLT3* expression benefit from sorafenib, which improves prognosis [34]. Therefore, the high expression of FLT3 promotes the proliferation and migration of HCC [35], which may be the reason for the high expression of FLT3 in vitro.

There were some limitations that need to be clarified in this study. First, patients with HCC were not stratified according to different primary factors (such as nonalcoholic fatty liver disease-associated hepatocellular carcinoma and hepatitis virus-associated hepatocellular carcinoma), and there might be necroptosis signature heterogeneity in different population. Second, bioinformatics provides an initial strategy for screening genes, but the function of these genes needs to be further explored via protein analysis and in vitro/vivo experiments.

## 5. Conclusion

This study established a valuable risk model based on necroptosis genes, which can effectively predict the prognosis of patients with HCC. Our results provided some potential biomarkers and targets, and further research will assist in elucidating the role of necroptosis genes in the progression of HCC.

## Figures and Tables

**Table 1:** Risk genes and coefficients based on TCGA-LIHC.

Gene	Coefficient
SQSTM1	0.19065746423157
FLT3	−0.441569284806212
HAT1	0.0328900450017848
PLK1	0.214656176920298
MYCN	0.218448389883388
KLF9	−0.013711346071549
HSP90AA1	0.131284235992642
TARDBP	0.31939225321784
TNFRSF21	0.0404023046649232

## Data Availability

Codes and other data used to support the findings of the study are available from the corresponding author upon reasonable request.

## Supplementary Materials

Supplementary Materials Figure S1. Relationship between hub genes and prognosis. (a) OS and (b) DFS outcomes using Kaplan–Meier curves. Figure S2. Validation of the LASSO risk model using the GSE54236 test cohort. (a) Distribution according to the risk scores. (b, c) High-risk patients have higher mortality rates. (d) ROC analysis. (e) PCA analysis. Supplementary Table 1. Necroptosis gene list. Supplementary Table 2. Gene primer. Supplementary Table 3. Total GSEA analysis results.

## References

[1] Frank D., Vince J. E. (2019). Pyroptosis versus necroptosis: similarities differences and crosstalk. *Cell Death & Differentiation*.

[2] Moriwaki K., Bertin J., Gough P. J., Orlowski G. M., Chan F. K. (2015). Differential roles of RIPK1 and RIPK3 in TNF-induced necroptosis and chemotherapeutic agent-induced cell death. *Cell Death & Disease*.

[3] Yatim N., Jusforgues-Saklani H., Orozco S. (2015). RIPK1 and NF-*κ*B signaling in dying cells determines cross-priming of CD8+ T cells. *Science*.

[4] Wu Y., Zhou B. P. (2009). Inflammation: a driving force speeds cancer metastasis. *Cell Cycle*.

[5] Liu Z. Y., Wu B., Guo Y. S. (2015). Necrostatin-1 reduces intestinal inflammation and colitis-associated tumorigenesis in mice. *American journal of cancer research*.

[6] Nie S., Huili Y., He Y., Hu J., Kang S., Cao F. (2022). Identification of bladder cancer subtypes based on necroptosis-related genes, construction of a prognostic model. *Frontiers in Surgery*.

[7] Wu Z., Huang X., Cai M., Huang P., Guan Z. (2022). Novel necroptosis-related gene signature for predicting the prognosis of pancreatic adenocarcinoma. *Aging (Albany NY)*.

[8] Sia D., Villanueva A., Friedman S. L., Llovet J. M. (2017). Liver cancer cell of origin, molecular class, and effects on patient prognosis. *Gastroenterology*.

[9] Seehawer M., Heinzmann F., D’Artista L. (2018). Necroptosis microenvironment directs lineage commitment in liver cancer. *Nature*.

[10] Pasparakis M., Vandenabeele P. (2015). Necroptosis and its role in inflammation. *Nature*.

[11] Zhao Z., Liu H., Zhou X. (2021). Necroptosis-related lncRNAs: predicting prognosis and the distinction between the cold and hot tumors in gastric cancer. *JAMA Oncology*.

[12] Song W., Ren J., Xiang R., Kong C., Fu T. (2021). Identification of pyroptosis-related subtypes, the development of a prognosis model, and characterization of tumor microenvironment infiltration in colorectal cancer. *OncoImmunology*.

[13] Liu S., Shao R., Bu X., Xu Y., Shi M. (2021). Identification of the pyroptosis-related gene signature for overall survival prediction in patients with hepatocellular carcinoma. *Frontiers in Cell and Developmental Biology*.

[14] Sung H., Ferlay J., Siegel R. L. (2021). Global cancer statistics 2020: GLOBOCAN estimates of incidence and mortality worldwide for 36 cancers in 185 countries. *CA: A Cancer Journal for Clinicians*.

[15] Engel J., Emeny R. T., Hölzel D. (2012). Positive lymph nodes do not metastasize. *Cancer and Metastasis Reviews*.

[16] Wang W., Kandimalla R., Huang H. (2019). Molecular subtyping of colorectal cancer: recent progress, new challenges and emerging opportunities. *Seminars in Cancer Biology*.

[17] Li X., Dong G., Xiong H., Diao H. (2021). A narrative review of the role of necroptosis in liver disease: a double-edged sword. *Annals of Translational Medicine*.

[18] Carloni V., Lulli M., Madiai S. (2018). CHK2 overexpression and mislocalisation within mitotic structures enhances chromosomal instability and hepatocellular carcinoma progression. *Gut*.

[19] Huntington J. T., Tang X., Kent L. N., Schmidt C. R., Leone G. (2016). The spectrum of E2F in liver disease-mediated regulation in biology and cancer. *Journal of Cellular Physiology*.

[20] Bisso A., Filipuzzi M., Gamarra Figueroa G. P. (2020). Cooperation between MYC and *β*‐catenin in liver tumorigenesis requires yap/taz. *Hepatology*.

[21] Wei S., Dai M., Zhang C. (2021). KIF2C: a novel link between Wnt/*β*-catenin and mTORC1 signaling in the pathogenesis of hepatocellular carcinoma. *Protein & Cell*.

[22] Reddy D., Kumavath R., Ghosh P., Barh D. (2019). Lanatoside C induces G2/M cell cycle arrest and suppresses cancer cell growth by attenuating MAPK, wnt, JAK-STAT, and PI3K/AKT/mTOR signaling pathways. *Biomolecules*.

[23] Pinter M., Jain R. K., Duda D. G. (2021). The current landscape of immune checkpoint blockade in hepatocellular carcinoma. *JAMA Oncology*.

[24] Datta M., Coussens L. M., Nishikawa H., Hodi F. S., Jain R. K. (2019). Reprogramming the tumor microenvironment to improve immunotherapy: emerging strategies and combination therapies. *American Society of Clinical Oncology Educational Book*.

[25] Yost K. E., Satpathy A. T., Wells D. K. (2019). Clonal replacement of tumor-specific T cells following PD-1 blockade. *Nature Medicine*.

[26] Zheng L., Qin S., Si W. (2021). Pan-cancer single-cell landscape of tumor-infiltrating T cells. *Science*.

[27] Goodall M. L., Fitzwalter B. E., Zahedi S. (2016). The autophagy machinery controls cell death switching between apoptosis and necroptosis. *Developmental Cell*.

[28] Carafa V., Nebbioso A., Cuomo F. (2018). RIP1-HAT1-SIRT complex identification and targeting in treatment and prevention of cancer. *Clinical Cancer Research*.

[29] Tian L., Yao K., Liu K. (2020). PLK1/NF-*κ*B feedforward circuit antagonizes the mono-ADP-ribosyltransferase activity of PARP10 and facilitates HCC progression. *Oncogene*.

[30] Deeraksa A., Pan J., Sha Y. (2013). Plk1 is upregulated in androgen-insensitive prostate cancer cells and its inhibition leads to necroptosis. *Oncogene*.

[31] Qin X.-Y., Suzuki H., Honda M. (2018). Prevention of hepatocellular carcinoma by targeting MYCN-positive liver cancer stem cells with acyclic retinoid. *Proceedings of the National Academy of Sciences*.

[32] Shi W., Feng L., Dong S. (2020). FBXL6 governs c-MYC to promote hepatocellular carcinoma through ubiquitination and stabilization of HSP90AA1. *Cell Communication and Signaling*.

[33] Stirewalt D. L., Radich J. P. (2003). The role of FLT3 in haematopoietic malignancies. *Nature Reviews Cancer*.

[34] Sun W., Li S.-C., Xu L. (2020). High FLT3 levels may predict sorafenib benefit in hepatocellular carcinoma. *Clinical Cancer Research*.

[35] Aydin M. M., Bayin N. S., Acun T., Yakicier M. C., Akçali K. C. (2016). Role of FLT3 in the proliferation and aggressiveness of hepatocellular carcinoma. *Turkish Journal of Medical Sciences*.

