# Supplementary material for: Identification and Validation of Prognosis-Related Necroptosis Genes for Prognostic Prediction in Hepatocellular Carcinoma
Source: J Oncol. 2022 Jun 29;2022:3172099. doi: 10.1155/2022/3172099 (PMC9259286; doi:10.1155/2022/3172099)
Supplement: Supplementary Materials — Figure S1. Relationship between hub genes and prognosis. (a) OS and (b) DFS outcomes using Kaplan–Meier curves. Figure S2. Validation of the LASSO risk model using the GSE54236 test cohort. (a) Distribution according to the risk scores. (b, c) High-risk patients have higher mortality rates. (d) ROC analysis. (e) PCA analysis. Supplementary Table 1. Necroptosis gene list. Supplementary Table 2. Gene primer. Supplementary Table 3. Total GSEA analysis results. [file 3172099.f1.zip › 3172099.f1/Supplementary table 1.docx]

**Supplementary table 1. Necroptosis genes list.**

| FADD |
| --- |
| FAS |
| FASLG |
| MLKL |
| RIPK1 |
| RIPK3 |
| TLR3 |
| TNF |
| TSC1 |
| TRIM11 |
| CASP8 |
| ZBP1 |
| MAPK8 |
| IPMK |
| ITPK1 |
| SIRT3 |
| MYC |
| TNFRSF1A |
| TNFSF10 |
| TNFRSF1B |
| TRAF2 |
| PANX1 |
| OTULIN |
| CYLD |
| USP22 |
| MAP3K7 |
| SQSTM1 |
| STAT3 |
| DIABLO |
| DNMT1 |
| CFLAR |
| BRAF |
| AXL |
| ID1 |
| CDKN2A |
| HSPA4 |
| BCL2 |
| STUB1 |
| FLT3 |
| HAT1 |
| SIRT2 |
| SIRT1 |
| PLK1 |
| MPG |
| BACH2 |
| GATA3 |
| MYCN |
| ALK |
| ATRX |
| TERT |
| SLC39A7 |
| SPATA2 |
| RNF31 |
| IDH1 |
| IDH2 |
| KLF9 |
| HDAC9 |
| HSP90AA1 |
| LEF1 |
| BNIP3 |
| CD40 |
| BCL2L11 |
| EGFR |
| DDX58 |
| TARDBP |
| APP |
| TNFRSF21 |
